# Prevalence and determinants of adequate postnatal care in Ethiopia: evidence from 2019 Ethiopia mini demographic and health survey

**DOI:** 10.1186/s12884-023-06147-7

**Published:** 2023-12-04

**Authors:** Mubarek Yesse Ashemo, Desalegn Shiferaw, Bayise Biru, Bikila Regassa Feyisa

**Affiliations:** 1 Department of Epidemiology, Faculty of Public Health, Jimma University, Jimma, Ethiopia; 2 Department of Public Health, College of Medical and Health Science, Werabe University, Werabe, Ethiopia; 3 Department of Public Health, College of Medical and Health Science, Dambi Dollo University, Dambi Dollo, Ethiopia; 4 Department of Human Nutrition and Dietetics, Faculty of Public Health, Jimma University, Jimma, Ethiopia; 5 Department of Public Health, Institute of Health, Wallaga University, Nekemte, Ethiopia

**Keywords:** Prevalence, Factors, Adequate postnatal care, Ethiopia

## Abstract

**Background:**

The postpartum period is critical for both the mother's and newborn child's health and survival. Rising morbidity and mortality are usually the consequence of absence of adequate, suitable, or timely care during that time period. There is lack of information on the adequacy of postnatal care in Ethiopia and this study was aimed to investigate adequacy of postnatal care and its determinants in the study area.

**Methods:**

In this study, we used a cross-sectional dataset from the 2019 Ethiopia Mini Demographic and Health Survey. A multistage stratified clustered design applied and survey weights were used to take into account the complicated sample design. A multilevel mixed effects logistic regression was fitted on 3772 women who were nested within 305 clusters. The fixed effect models were fitted and expressed as adjusted odds ratios with 95% confidence intervals, while intra-class correlation coefficients, median odds ratio, and proportional change in variance explained measures of variation. As model fitness criteria, the deviance information criterion and the Akaike information criterion were used.

**Results:**

This study found that only 563(16.14%, 95% CI: 16.05–16.24) women had adequate post natal care. Age of between 25–35 years old (AOR = 1.55, 95%CI = 1.04–2.31), secondary level of education (AOR = 2.23, 95%CI = 1.43–3.45), Having parity of between two and four had (AOR = 0.62, 95%CI = 0.42 0.93), having ANC follow up four and above (AOR = 1.74, 95%CI = 1.31–2.33), being residents of Oromia region (AOR = 0.10, 95CI = 0.02- 0.43) were strong predictors of adequate postnatal care.

**Conclusion:**

The study found that prevalence of adequate PNC in Ethiopia was significantly low. To increase postnatal care adequacy, it was recommended to reinforce existing policies and strategies such as increasing number of antenatal care follow up, and scheduling mothers based on the national postnatal care follow-up protocol.

## Introduction

Maternal and child mortality rates are frequently used to assess the performance of a country's health system, and high rates have continuously been considered to be among the most significant health challenges, which persist in developing nations today. The likelihood of a mother and child dying after delivery is still high despite the expansion of maternal and child health programs and global maternal health communities in the most vulnerable areas [1–6]. There is an increasing number of evidence as well as recommendations emphasizing the importance of providing high-quality, cost-effective care during this time [3, 4, 7]. As a result, inadequate care during the postnatal period may lead to mortality or morbidity, as well as missed opportunities to engage in various healthy behaviors that advantage the mother and her new-born baby [8].

The postpartum period is critical for both the mother's and newborn’s health and survival [9–11]. Maternal and newborn morbidity and mortality during the postpartum period are usually related to inadequate and inappropriate care during that time [12]. Evidently, more than half of all neonatal deaths happened during the first two days of life, with three-quarters occurring during the first week of life [13]. Similarly, 45% of postpartum maternal deaths happened within 24 h of delivery, and the risk continues into the second week [14].

More than 75% of all neonatal deaths occurred due to Preterm birth complications such as, birth asphyxia, and sepsis while postpartum haemorrhage, hypertensive disorders, and infections account for the majority of maternal deaths [13]. All of these are preventable and controllable causes, implying that high-quality care during labor and delivery, as well as skilled care and therapy in the early postpartum period, may help to reduce this fatality [15]. As a result, international initiatives have been geared in that direction. One of the most important programs for improving mother and child health is postnatal care (PNC), which consists of a set of services provided to the mother and newborn starting immediately after the placenta is delivered and persisting for the first 42 days after birth [16]. In fact, postnatal visits give medical professionals the chance to encourage healthy habits like breastfeeding, proper cord care, and hand washing check for danger signs, and keep track of the mother's and newborn's development, and general health [12]. Additionally, it allows for the early identification and treatment of issues related to labor and delivery, as well as counseling and advanced care referrals when necessary [12].

Despite the fact that the world's mortality rates have been declining, the majority of maternal deaths and the highest rates of neonatal deaths continue to occur in low- and middle-income nations, particularly those in sub-Saharan Africa [5, 16]. Although the Ethiopian government and its partner have made tremendous efforts to increase the utilization of maternal health services, the postnatal care coverage still remains low in the country. Ethiopia is dedicating considerable resources towards mitigating the mortality rates of both mothers and newborns, with the aim of achieving the target outlined in the Sustainable Development Goals by the year 2030. While it is true that there has been a decline in maternal and infant mortalities within Ethiopia from the years 2000 to 2016, the current mortality rates remain at an unacceptably high level, necessitating further action [17].

A cross sectional study conducted from review of 2016 Rewanda demographic and health survey in showed that only 44.3% newborns got all of the five recommended postnatal care services [18]. A cross sectional study conducted in a northern Ethiopia showed that the prevalence of utilization of at least one PNC was only 24.6% [19]. A cross sectional study conducted in Northern Ethiopia showed that on 28.4% of the mothers took the recommended number of postnatal care visits [20]. The analysis of EDMHS, 2019 revealed that only 33.7% women had taken the recommended number of PNC [21]. The spatial analysis of 2016 EDHS data revealed that the PNC utilization coverage in Ethiopia is very low. The analysis found that only 6.9% women utilized PNC with in the first six weeks of delivery [22]

The analysis of large community based cross-sectional studies in Ethiopia showed that PNC utilization is affected by factors at both the individual and community levels [22, 23]. Other many studies showed that utilization of postnatal care is associated with residence, maternal education, ANC during pregnancy, type delivery, place of birth, complications during labor, receiving counsel during antenatal care visits, wealth index, parity, lack of information about postnatal care, and cultural and religious beliefs [12, 18–20, 23–34].

Despite few studies conducted to reveal the prevalence of taking adequate number of PNC, there has been no study done to date to reveal the prevalence of adequate postnatal care content and its associated factors in Ethiopia. Therefore, this study is aimed to provide evidence about prevalence of adequate postnatal care content and its associated factors in Ethiopia.

## Methods and materials

### The study design and stetting

The study was conducted in Ethiopia. Ethiopia is a landlocked nation in East Africa that borders Eritrea to the north, Sudan and South Sudan to the west, Somalia and Djibouti to the east, and Kenya to the south. Ethiopia's population is estimated to be 126 million people, with approximately 25 million women of reproductive age. Ethiopia is divided administratively into 11 regions and two city administrations [35]. Human resources for health, access to essential maternal health care, and a lack of infrastructure are major challenges in Ethiopian maternal and child health care [36].

### Study participants

In this study, women who had given birth within the five years prior to the survey and underwent at least one postnatal checkup, either before being discharged from the hospital after giving birth or after having given birth at home and being discharged from the hospital were included. All the reproductive age women (15–49 years) found in the selected clusters at least one night before the data collection during the study period. All reproductive age women were taken as source population and reproductive age women in the selected clusters as a study population while those eligible women who were included in 2019 Ethiopian Mini Demographic and Health Survey (EMDHS) enumeration areas (EAs) at least one night before data collection were taken as sample population.

### Sample size determination, sampling technique and procedure

**Fig. 1:**
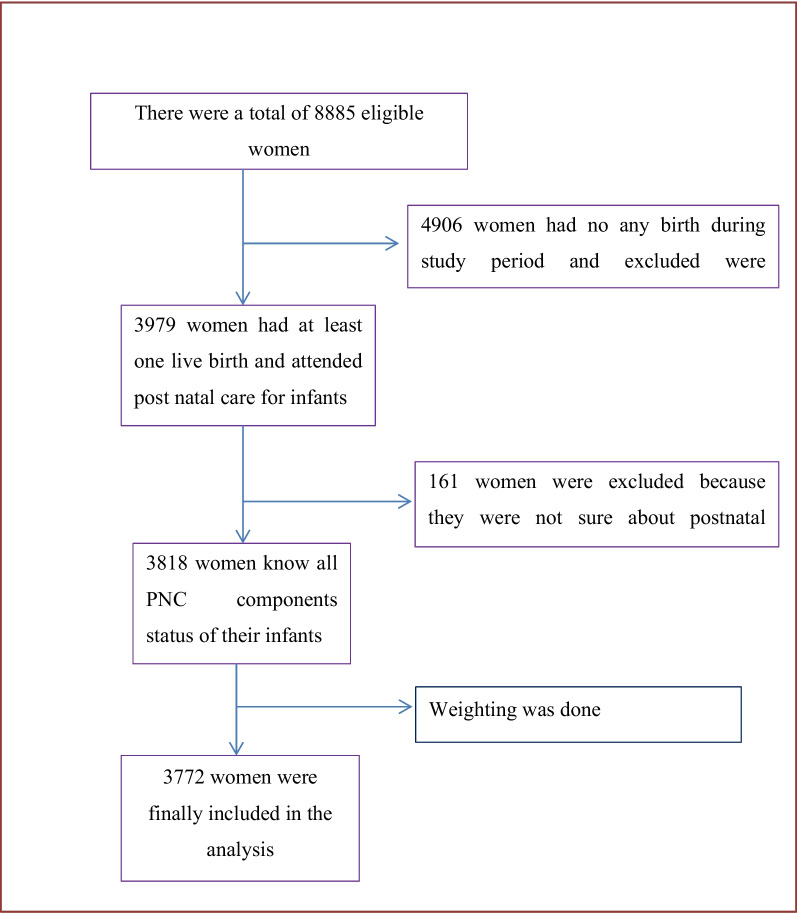
Shows sampling procedure and final sample sized used for the study

In this study a multistage stratified sampling technique was used. The full list of 149,093 enumeration areas (EAs) made for the 2019 Ethiopia Population and Housing Census (PHC) served as the sampling frame. An EA is a region that typically encompasses 131 HHs. There were 21 sampling strata created by dividing each region and administrative city into urban and rural areas. In two stages, independent selections of EA samples were made in each stratum. In the first stage, 305 EAs (93 in urban areas and 212 in rural areas) were chosen independently in each sampling stratum with a probability proportional to the size of the EAs. All of the chosen EAs underwent an HH listing operation, and the resulting list of HHs served as a sampling frame for the subsequent stage. In the second stage of selection, a fixed number of 30 HHs were chosen from the newly created HH listing with an equal probability of systematic selection. From each EA household with women who gave birth five or less than five years prior to study were randomly selected and one woman was selected randomly if the selected house had had more than one eligible woman during the study period. For the analysis, weighted data was used (Fig. 1).

### Data collection procedure

The dataset utilized in this study was obtained from the DHS program accessed from https://dhsprogram.com/Data/terms-of use.cfm. The Ethiopian Public Health Institute (EPHI) carried out the 2019 Ethiopia Mini Demographic and Health Survey (2019 EMDHS) in collaboration with the Central Statistical Agency (CSA) and the Federal Ministry of Health (FMoH), under the overall supervision of the Technical Working Group (TWG). The duration of data collection was between March and June of 2019. The World Bank, the United States Agency for International Development (USAID), and the United Nations Children's Fund provided funding for the 2019 EMDHS (UNICEF). The DHS Program, a USAID-funded project that provides support and technical assistance in the implementation of population and health surveys in countries around the world, was where ICF provided technical assistance. In this study, a community-based cross-sectional survey was conducted from March 21, 2019, to June 28, 2019, among women of reproductive age group, based on a nationally representative sample that provided estimates at the national and regional levels and for urban and rural areas.

## Variables

### Dependent variable

#### Adequate Postnatal care

The postnatal care content was the outcome variable. Based on WHO recommendations [7] and the availability of data in the 2019 EMDHS dataset, adequate PNC content was defined as a woman receiving all five PNC components, which included having the cord examined, measuring the baby's temperature, counseling on newborn danger signs, counseling on breastfeeding, and having an observed breastfeeding session. Women who had taken fewer than five of the components were considered to have received inadequate postnatal care (“labeled as 0”), while those who had taken five of the five components of PNC were taken as having received adequate PNC (“labeled as 1”) [18]. The women provided this information on their own.

### Independent variables

#### Individual level (Level I) variables

Age, Sex of the house head, Marital status, Occupation, Religion, Educational status, Ethnicity, Parity, Family size, Number of ANC follow up, Place of delivery, Prenatal health extension workers visit, and Wealth index.

#### Community level (Level II) variables

Region and Residence.

#### Data processing and analysis

STATA 14 was used for all statistical analyses. In order to account for the uneven stratification of different regions, the use of multistage sampling to enlist participants, and the ability to generalize the results to the national reference population, we conducted a weighted analysis. Categorization was performed on continuous variables, and categorical variables were recategorized. Prior to analytic analysis, descriptive analysis was conducted and presented using percentages and frequencies. Because the current data was correlated at the cluster level, the random effect for predictors of adequate PNC among Ethiopian reproductive age women was used as a cluster number/Enumeration Area. In the same cluster, it was assumed that prevalence of adequate PNC is constant. Therefore, multilevel mixed effects logistic regression was applied in this study. In order to determine the relationship between the independent variables and the dependent variable, bi-variable two-level mixed-effects logistic regression analyses were first conducted. The final model of the multivariable two-level mixed-effect logistic regression model, in which odds ratio with 95% confidence intervals were estimated to identify the independent variables of adequate PNC, included the overall categorical variables with a p value of 0.20 at the bivariate two-level mixed-effect logistic regression analysis. To declare statistical significance, P-values lower than 0.05 were used. Four models—the null model (model with no factors), model I (model with only maternal factors), model II (model with only community factors), and model III (model with both maternal and community level factors—are shown in this analysis. The percentage of variation between clusters in the overall variation was used to calculate the intra-class correlation (ICC). Models were compared using Akaike information criteria (AICs), Bayesian information criterion (BICs), and deviance value (see Table 3).

## Results

### Socio-demographic characteristics of study particapants

In this study a total weighted sample of 3772 women were included. About half (50.55%) women were at the age of between 25–35 years, had delivered in health facility (51.14%), and had primary education (51.75%). The majority of women (93.40%) were married, (86.63%) had male household head, delivered by cesarean Sect. (93.88%), and had reported prenatal health extension workers visit (83.18%). About three fourth (73.87%) of the participants were rural residents, and large number (39.44%) of women were residents of Oromia region. More than one third (35.95%) of women were orthodox religion followers. Less than half (45.26%) had family size of five to seven, had parity between two to four (44.21%), and more than half (57.47%) of women had less than four ANC contacts. About one fifth (21.22%) of the women had poorest wealth index (Table 1).

**Table 1:** Socio-demographic and reproductive characteristics of mothers who utilized postnatal care in Ethiopia, 2019 EMDMS

Variables (*n* = 3772)	Category	Frequency	Percent
**Age**	15–24 years	950	25.19
	25–35 years	1907	50.55
	35–49 years	915	24.26
**Marital status**	Never married	20	0.54
	Married	3523	93.40
	Living together	23	0.62
	Divorced	116	3.08
	Widowed	39	1.02
	Separated	51	1.34
**Educational status**	No education	1952	51.75
	Primary education	1346	35.68
	Secondary education	327	8.68
	Higher Education	147	3.89
**Residence**	Urban	986	26.13
	Rural	2786	73.87
**Region of residence**	Tigray	267	7.07
	Afar	50	1.31
	Amhara	758	20.09
	Oromoia	1487	39.44
	Somali	216	5.73
	Benshangul	46	1.21
	SNNPR	780	20.68
	Gambella	18	0.5
	Harari	10	0.27
	Addis Ababa	120	3.17
	Dire Dawa	20	0.53
	Tigray	267	7.07
**Religion**	Orthodox	1356	35.95
	Catholic	13	0.34
	Protestant	1065	28.25
	Muslim	1287	34.11
	Tradition	44	1.16
	Other	7	0.19
**Parity**	1	776	20.58
	2–4	1668	44.21
	5 and above	1328	35.21
**Sex of the house head**	Male	3268	86.63
	Female	504	13.37
**Family size**	1–4	1237	32.78
	5–7	1707	45.26
	8 and above	828	21.96
**Wealth index**	Poorest	800	21.22
	Poorer	789	20.91
	Middle	737	19.55
	Richer	683	18.11
	Richest	762	20.21
**Number of ANC**	< four	2176	57.47
	Four and above	1596	42.23
**Place of Delivery**	Home	1843	48.86
	Health facility	1929	51.14
**Delivery by caesarian section**	No	3541	93.88
	Yes	231	6.12
**Prenatal health extension workers visit**	No	3137	83.18
	Yes	635	16.82

### Postnatal care contents utilization

**Fig. 2:**
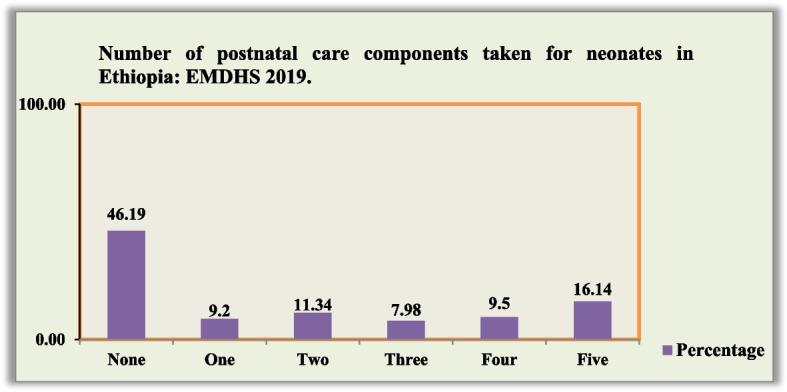
Proportion of number of postnatal care contents utilization among mothers who utilized postnatal care in Ethiopia, 2019 EMDMS

Concerning PNC content, 423 (11.2%, 95% CI: 10.1–12.3) of the 3772 women had received all postnatal care components, with the counseled on newborn danger signs (35.85%) was relatively more received PNC component (Table 2 and Fig. 2).

**Table 2:** Postnatal care contents utilization among mothers who utilized postnatal care in Ethiopia, 2019 EMDMS

Variables (*n* = 3772)	Category	Frequency	Percent
**Postnatal care content utilized**	Cord examined	994	26.34
	Temperature Measured	991	26.26
	Counseling on newborn dangers	778	20.64
	Counseling on breast feeding	1352	35.85
	Breast feeding session observed	1237	32.80

### Prevalence of adequate postnatal care

**Fig. 3:**
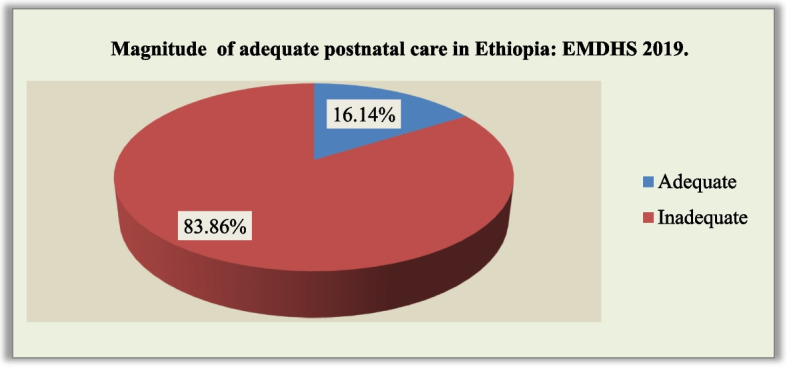
Prevalence of adequate postnatal care in Ethiopia, from 2019 Ethiopian mini demographic and health survey data

Prevalence of adequate PNC was 563(16.14%, 95% CI: 16.05–16.24) (Fig. 3).

### Factors associated with adequacy of postnatal care in Ethiopia

In this study, independent variables were measured to investigate their relationship with the outcome variable (adequate postnatal care). The association between each variable and the outcome variable was investigated using univariable and multilevel logistic regression model. The first step during analysis was selection of best model that fit the data and checking the general assumptions of the model. After selecting the best model, both univariable and multivariable multilevel logistic regression model analysis were conducted.

### Model selection

In this study, a two-level mixed-effects logistic regression model was used to examine the effects of community characteristics and individual-level factors on adequacy of postnatal care service utilization. According to Table 3, Model 0, which was fitted with random intercept, was better than ordinary logistic regression (OLS) which was fitted without random intercept. Model one was better than null model (model 0). In model 0, effect of the intra-class correlation coefficient (ICC) estimated the community variance in adequacy of PNC at 46.0%. This means that unobserved community characteristics account for 46.0% of the residual variation in the propensity to have adequate PNC.

**Table 3:** Random effects on postnatal check-ups in Ethiopia: A multilevel logistic regression model

	**OLR Model**	**Model 0**	**Model 1**	**Model 2**	**Model 3**
ICC	With no ICC	46.0.0%	30.13%	35.15%	25.54%
Log likelihood	-1593.467	1141.415	-986.0709	-1104.369	-964.1862
AIC	3188.934	2286.831	2022.142	2234.737	2000.372
BIC	3195.182	2299.326	2178.329	2315.954	2225.282

To estimate the associations between individual and community variables and the likelihood of having adequate postnatal care at a health facility, three multilevel random intercept logistic regression models were fitted. The first model only included individual-level variables, the second model only included community-level variables, and the final model (full) includes both individual and community-level variables (Table 3).

In this study result and discussion elaboration was provided for association which was obtained after adjusting individual level factors with community level factors (model III). Women who were at the age of between 25–35 years had one and half (AOR = 1.55, 95%CI = 1.04–2.31) more odds of having adequate PNC when compared to women who were at age of 15–24 years. Women who attended secondary level of education had almost two times (AOR = 2.23, 95%CI = 1.43–3.45) more odds of having adequate PNC when compared to women who did not attend any education. Women who had parity of between two and four had 38% (AOR = 0.62, 95%CI = 0.42 0.93) lesser odds of having adequate PNC when compared to women who had parity of 5 and above. In this study, women who had ANC follow up four and above had almost two times (AOR = 1.74, 95%CI = 1.31–2.33) higher odds of having adequate PNC when compared to women who had ANC follow up of less than four times. Women who were residents of Oromia region had 90% (AOR = 0.10, 95CI = 0.02- 0.43) less odds adequate PNC when compared to women who were residents of Gambella region. Women who gave birth at health facility had twelve times (AOR = 12.43, 95CI% = 7.8–19.92) more odds of adequate PNC, and women who delivered by cesarean section had almost one and half times (AOR = 1.61, 95%CI = 1.08–2.39) higher odds of PNC when compared to their counterparts (Table 4).

**Table 4:** Multilevel mixed-effect logistic regression results of maternal and community-level factors associated with adequacy of postnatal care in Ethiopia, 2019 EMDHS

Variables (*n* = 3818)	Category	Model I	Model II	Model III
		**AOR (95%CI)**	**AOR (95%CI)**	**AOR (95%CI)**
**Age**	15–24 years	Reference	Reference	Reference
	25–35 years	1.56(1.05–2.31)^*^		1.55(1.04–2.31)^*^
	35–49 years	1.20(0.72–2.0)^¶^		1.20(0.72–2.01)^*^
**Marital status**	Never married	Reference		Reference
	Married	1.85(0.29–11.71)^¶^		2.13(0.31–14.24)^¶^
	Living together	6.92(0.80–60.05)^*^		7.95(0.88–72.10)^¶^
	Divorced	0.70(0.04–11.63)^¶^		0.83(0.05–14.28)^¶^
	Widowed	1.81(0.25–13.0)^¶^		2.12(0.28–15.93)^¶^
	Separated	1.64(0.18–14.23)^¶^		1.68(0.18–15.21)^¶^
**Educational status**	No education	Reference		Reference
	Primary education	0.97(0.70–1.35)^¶^		0.96(0.69–1.33)^¶^
	Secondary education	2.33(1.50–3.40)^Φ^		**2.23(1.43–3.45)**^**¶**^
	Higher Education	1.09(0.59–1.99)^¶^		0.92(0.50–1.70)^¶^
**Residence**	Urban		1.66(0.89–3.09)^¶^	0.81(0.43–1.53)^¶^
	Rural		Reference	Reference
**Region of residence**	Tigray		0.83(0.18–3.74)^¶^	0.41(0.10 -1.77)^¶^
	Afar		0.29(0.05–1.73)^¶^	0.52(0.09–3.06)^¶^
	Amhara		0.22(0.05–0.98)^*^	0.16(0.09–3.06)^¶^
	Oromoia		0.13(0.03–0.59)^*^	0.10(0.02- 0.43)^*^
	Somali		0.10(0.02–0.50)^*^	0.22(0.04 -1.09)^¶^
	Benshangul		0.91(0.17–4.90)^¶^	0.48(0.09- 2.47)^¶^
	SNNPR		0.34(0.07–1.50)^¶^	0.23(0.01- 2.11)^¶^
	Gambella		Reference	Reference
	Harari		0.15(0.01–2.73)^¶^	0.12(0.01–2.11)^¶^
	Addis Ababa		2.27(0.48–10.60)^¶^	1.25(0.28–5.58)^¶^
	Dire Dawa		0.25(0.29–2.07)^¶^	0.15(0.02- 1.22)^¶^
**Sex of the house head**	Male	Reference		Reference
	Female	0.80(0.53–1.21)^¶^		0.73(0.48–1.11)^¶^
**Family size**	1–4	Reference	Reference	Reference
	5–7	1.12(0.70–1.79)^¶^		1.07(0.67–1.72)^¶^
	8 and above	1.55(10.4–2.30)^*^		1.47(0.99- 2.19)^¶^
**Place of Delivery**	Home	Reference		Reference
	Health facility	13.0(8.09–20.92)^Φ^		**12.43(7.8–19.92)**^Φ^
**Parity**	1	0.54(0.31–0.93)^*^		0.55(0.31- 0.96)^*^
	2–4	0.62(0.42–0.92)^*^		0.62(0.42 0.93)^*^
	5 and above	Reference		Reference
**Wealth index**	Poorest	Reference		Reference
	Poorer	1.00(0.59–1.67)^¶^		1.09(0.64–1.86)^¶^
	Middle	0.96(0.56–1.64)^¶^		1.08(0.62–1.86)^¶^
	Richer	0.93(0.54–1.61)^¶^		1.01(0.58–1.78)^¶^
	Richest	1.25(0.69–2.27)^¶^		1.02(0.51–2.00)^¶^
**Number of ANC**	< four	Reference		Reference
	Four and above	1.77(1.33–2.35)^Φ^		**1.74(1.31- 2.33)**^Φ^
**Delivery by CS**	No	Reference		Reference
	Yes	1.63(1.10–2.42)^*^		1.61(1.08–2.39)^*^
**Prenatal HEWs visit**	No	Reference		Reference
	Yes	0.99(0.67–1.46)^¶^		1.07(0.72–1.57)^¶^

**Key:** ^¶^ = *P* > 0.05, ^*^ = *P* ≤ 0.05, ^Φ^ = *P* < 0.001

## Discussion

Identifying maternal characteristics and community-level determinants of postnatal care utilization can help to design various interventions to improve maternal and child health. As a result, multilevel mixed effect modeling was used in this study to identify maternal and community-level determinants of postnatal care check-ups in Ethiopia.

According to this study finding, 563(16.14%) of the mothers had received all five PNC components, with cord examination, having breastfeeding sessions observed, and breastfeeding counseling being the most frequently reported (35.85%), but with less reporting (20.64%) for counseling on newborn dangers. The observed prevalence of adequate PNC use remains insufficient to achieve the desired reduction in postnatal-related child and maternal mortality and morbidity. This could be explained in part by a lack of community awareness about PNC components in Ethiopia or by the lower emphasis placed on some PNC components by healthcare/PNC providers. As a result, there is a need for tailored ANC education to raise mothers' awareness of PNC contents, as well as ongoing medical education for the various PNC providers. Nonetheless, such low rates of overall adequate PNC and imbalances in receipt of specific PNC components have been reported in other resource-constrained countries, including Tanzania [26], Uganda [29], Nigeria [25], Zambia [30], Nepal [37], Bangladesh [38], and rural China [39] and Rewanda [18].

In this study, women who had a caesarean delivery had more odds having adequate PNC when compared to mothers who had another mode of delivery. This is consistent with studies conducted in Ethiopia [24] and in rural Tanzania that found cesarean section delivery was positively associated with postnatal care use [26]. Women who have had a cesarean section may stay at the facility for about two days and receive a postnatal check-up.

Parity was also found to affect receipt of adequate PNC, with women with parity of between two and four having a 38% lower chance of receiving adequate PNC than those with parity of 5 and above. Similar study conducted in Rewanda revealed concurrent finding [18]. This could be because women with multiple parities have more experience with the importance of ANC and PNC from previous pregnancies and births than women giving birth for the first time [12]. It differs from several other studies that found first-time mothers to be more likely to use and visit PNCs [20, 25, 31, 37, 40]

This study also found that women who had more frequent ANC are more likely to have higher odds of PNC than women who had lesser frequency of ANC follow up. Similar finding was reported in studies conducted in Ethiopia [41] and Tanzania [26]. ANC visits are also meant to educate, brief, and counsel the mother on how to prepare for delivery as well as what to expect during the postpartum and postnatal period [42, 43]. This implies that mothers with lower ANC frequency are more likely to have missed key PNC information, such as its importance, contents, and when and where to receive it, leaving them with fewer chances of receiving adequate PNC [12, 30].

Women who were residents of Oromia region were less likely to have adequate PNC than women in the Gambella region. This finding is surprising given that the Gambella is a poor and pastoral region in Ethiopia, with the majority of its residents living in rural areas and being one of the regions with the lowest level of education in the country [44]. However, the possible cause of this discovery is unknown and we propose that additional research be conducted to investigate the reasons for this finding. Previous research has also found a link between region of residence and utilization of postnatal care services [24, 25, 27, 33]. When compared to those with higher education, women with secondary education had higher odds of adequate PNC. In contrast to this finding, most previous studies show that use of PNC services increases with higher levels of education [25, 28, 30, 45, 46].

In terms of place of delivery, mothers who delivered at health facility had more odds of having adequate PNC when compared to those who delivered at home. This finding is consistent with previous research in Ethiopia [34], Zambia [47], and Nepal [32]. This could be because women who gave birth in a health facility have more opportunities for health education related to PNC services during their delivery and thus have access to learning about the types, benefits, and availability of PNC services during their stay in the health facility [48].

The odds of adequate PNC higher among mothers who are at age of between 25–34 years when compared to mothers who are below or above this age group. This finding was supported by the study conducted in Malawi [31]. This might due to the reason that women during their age of 25–34 are more active and autonomous. Moreover, they may have some experience of birth but may not be very bored of large number of family size during this age.

### Limitations and strengths of the study

We estimated the effects of contextual variables and their interactions with individual-level variables on the use adequacy of PNC in Ethiopia using advanced analytic techniques and multilevel cluster-level random effect models, which is critical for developing effective strategies to improve adequate utilization of PNC in the country. Despite efforts to reduce biases through the use of memory aids, pretesting survey tools, training data collectors, and allocating adequate days for primary data collection, the findings of this study may have been influenced by social desirability and recall biases. Another important limitation of this study is that health facility and health professional related factors that could have influence the outcome variable did not included in the analysis because they were not captured by the 2019 EMDHS data.

### Conclusion and recommendations

The study found that prevalence of adequate PNC in Ethiopia was considerably low. Adequacy of PNC was significantly associated with maternal educational status, parity, and mode of delivery, ANC follow up, place of delivery, age of mothers and region of residence. To increase postnatal care adequacy, it was recommended to reinforce existing policies and strategies such as increasing number of antenatal care follow up, and scheduling mothers based on the national postnatal care follow-up protocol. Exploring women's perspectives on adequate PNC services in general, as well as the acceptability and feasibility of keeping women and newborns in health facilities for at least 24 h for postpartum observation, would be beneficial.

## Data Availability

Data sets used and analyzed during the current study are available from the corresponding author on reasonable request.

## Abbreviations

ICC: Intra-class correlation
AIC: Akaike information criteria
BIC: Bayesian information criterion
ANC: Ante natal care
EMDHS: Ethiopian Mini Demographic and Health Survey
DHS: Demographic and Health Survey
PNC: Post natal care
AOR: Adjusted odds ratio
WHO: World health organization
